# To Whom It May Concern

**Published:** 1869-11

**Authors:** C. B. Butler, W. P. Horton

**Affiliations:** Pres’t. O. S. D. Society


### TO WHOM IT MAY CONCERN.

The law entitled " An Act to Regulate the Practice of Dentistry in the State of Ohio," passed May 8th, 1868, provides in Section 1st, that any person commencing the practice of Dentistry in this State, after the date above mentioned, shall have a diploma from some Dental college, or a certificate of qualification from the State Dental Society; and Section 2d imposes a fine of from fifty to two hundred dollars for each violation of the provisions of this Act. Now, therefore, this is to notify all persons interested that the Board of Examiners of the Ohio State Dental Society will meet at the Neal House, in the city of Columbus, on Wednesday the 8th day of December, 1869, at 12 o'clock M., for the examination of all such persons as are desirous of commencing, or have commenced the practice of Dentistry, since the passage of the Act herein mentioned.

C. R. BUTLER, D. D. S., Cor. Sec.

